# Impacts of polymorphisms in drug-metabolizing enzyme and transporter genes on irinotecan toxicity and efficacy in Thai colorectal cancer patients

**DOI:** 10.1371/journal.pone.0338442

**Published:** 2025-12-12

**Authors:** Natthakul Akarapredee, Chalirmporn Atasilp, Chonlaphat Sukasem, Pimonpan Jinda, Rattanaporn Sukprasong, Jiraporn Jensuriyarkun, Soravit Wongjitjanyong, Patompong Satapornpong, Natchaya Vanwong

**Affiliations:** 1 The M.Sc. Program in Clinical Biochemistry and Molecular Medicine, Department of Clinical Chemistry, Faculty of Allied Health Sciences, Chulalongkorn University, Bangkok, Thailand; 2 Chulabhorn International College of Medicine, Thammasat University, Pathum Thani, Thailand; 3 Department of Pathology, Division of Pharmacogenomics and Personalized Medicine, Faculty of Medicine Ramathibodi Hospital, Mahidol University, Bangkok, Thailand; 4 Laboratory for Pharmacogenomics, Clinical Pathology, Ramathibodi Hospital, Bangkok, Thailand; 5 Division of General Pharmacy Practice, Department of Pharmaceutical Care, College of Pharmacy, Rangsit University, Pathum Thani, Thailand; 6 Excellence Pharmacogenomics and Precision Medicine Centre, College of Pharmacy, Rangsit University, Pathum Thani, Thailand; 7 Clinical Chemistry, Faculty of Allied Health Sciences, Chulalongkorn University, Bangkok, Thailand; Shoklo Malaria Research Unit, THAILAND

## Abstract

**Introduction:**

Irinotecan is a chemotherapy agent commonly prescribed for metastatic colorectal cancer but often leads to neutropenia. Variations in genes encoding drug-metabolizing enzymes and transporters may affect the toxicity and effectiveness of irinotecan. This study aimed to examine the impact of these genetic polymorphisms on irinotecan outcomes in Thai colorectal cancer patients.

**Methods:**

The study retrospectively analyzed 41 metastatic colorectal cancer patients treated with irinotecan-based chemotherapy. Genotyping was conducted for 23 single nucleotide polymorphisms in genes including *UGT1A1, CYP3A4, CYP3A5, CES1, ABCB1, ABCC2, ABCC5, ABCG1, ABCG2,* and *SLCO1B1*.Toxicity and efficacy were assessed, with statistical significance set at a Bonferroni-corrected P value < 0.002.

**Results:**

In terms of toxicity, UGT1A1*6 was significantly associated with both all-grade and severe neutropenia in the first cycle (*p* < 0.001) and severe neutropenia in the second cycle (*p* < 0.002). Lower absolute neutrophil count was observed among intermediate and poor UGT1A1 metabolizers (*p *< 0.001). The *ABCC2* -24C > T variant was linked to all-grade neutropenia in the second cycle (*p *= 0.001). For efficacy, patients with the wild-type UGT1A1*6 had longer progression-free survival (PFS) (*p* < 0.002). Additionally, the *SLCO1B1 521T > C* variant was associated with improved PFS (*p* < 0.002).

**Conclusion:**

UGT1A1*6 and *ABCC2* -24C > T variants emerge as potential predictors of irinotecan-induced neutropenia, while UGT1A1*6 and *SLCO1B1 521T > C* may serve as markers of prolonged PFS in Thai patients. Validation through larger prospective studies is essential to confirm and refine these genetic associations.

## Introduction

Colorectal cancer (CRC) ranks as the third most common cancer globally and is the second leading cause of cancer-related deaths [1]. In Thailand, CRC represents about 10.3% of newly diagnosed cancer cases and is currently the third most prevalent cancer in the population [2]. Irinotecan (CPT-11) has been approved for the treatment of advanced or metastatic colorectal cancer, either as a standalone therapy or, more commonly, as part of combination chemotherapy regimens [3]. However, irinotecan treatment is frequently associated with severe toxicities, such as neutropenia and diarrhea, which can lead to treatment interruptions or discontinuation, potentially compromising the patient’s prognosis and quality of life [4].

The uptake and transport of irinotecan into the liver are facilitated by transporters such as SLCO1B1, ABCB1, ABCC2, ABCC5, ABCG1 and ABCG2 [5–7]. Irinotecan is metabolized in the liver, where it undergoes hydrolysis by carboxylesterases (CES) to produce SN-38, its active metabolite and a potent inhibitor of Topoisomerase I [5]. SN-38 targets Topoisomerase I, blocking DNA replication in cancer cells, which ultimately leads to cell death. SN-38 is subsequently glucuronidated to form SN-38 glucuronic acid and is detoxified in the liver through conjugation by the UGT1A1 family, resulting in the release of SN-38G into the intestines for elimination [5]. At the same time, the SN-38 oxidation pathway, mediated by the P450 CYP3A4/5, competes with the activation and detoxification pathways of irinotecan. This oxidation of irinotecan leads to the formation of inactive metabolites [5]. Both CYP3A4/5 and UGT1A1 play crucial roles in the elimination of irinotecan by regulating the quantity and timing of the active product, SN-38.

Previous studies have highlighted the complex pharmacogenetics of irinotecan, with a particular focus on *UGT1A1*. Genetic polymorphisms in *UGT1A1*, particularly UGT1A1*28 and UGT1A1*6, are associated with reduced enzyme activity [8], leading to the accumulation of SN-38 and an increased risk of adverse drug reactions related to irinotecan treatment [9,10]. Besides *UGT1A1*, other drug-metabolizing enzymes and transporter genes may affect the efficacy and toxicity of irinotecan. CYP3A and CES, both involved in irinotecan metabolism, can influence SN-38 plasma concentrations, potentially impacting the drug’s efficacy and toxicity [11]. Since CYP3A4/5 plays a key role in irinotecan’s hepatic metabolism, genetic polymorphisms may contribute to interindividual variability in metabolism, efficacy, and toxicity. The CYP3A5*3 allele, which causes alternative splicing and protein truncation, results in a lack of CYP3A5 expression in the liver [12]. In contrast, the CYP3A4*1B variant is associated with increased CYP3A4 expression [13]. Additionally, polymorphisms in the *CES1* gene have been linked to adverse events in patients receiving irinotecan treatment [14].

Irinotecan and its active metabolite, SN-38, are substrates of ABC transporters, and polymorphisms in these transporters may affect irinotecan pharmacokinetics [15], as well as its efficacy and associated toxicities [5,16]. For instance, individuals carrying the *ABCB1* SNP (rs1045642) showed a higher risk of early toxicity and reduced treatment response [17]. Additionally, carriers of the *ABCB1* haplotype (including rs1045642, rs1128503, rs2032582) experienced lower response rates and shorter survival [17]. Moreover, polymorphisms in *ABCC1* and *ABCC2* (rs3740066), as well as *ABCG2* (rs2231137), were found to independently predict toxicities, such as grade 3 diarrhea [18]. Specifically, the *ABCC2* variant (rs3740066) is significantly associated with grades 1–4 neutropenia during the first treatment cycle in the Thai population treated with irinotecan [9]. OATP1B1, which is encoded by the SLCO1B1 gene, plays a role in the hepatic uptake of SN-38. The SLCO1B1*1b variant may serve as a protective biomarker against neutropenia and could enhance efficacy [19,20]. Conversely, the SLCO1B1*5 variant (rs4149056) is associated with decreased transporter activity, resulting in higher SN-38 plasma concentrations and an elevated risk of neutropenia, particularly in combination with UGT1A1*28 variant alleles [20].

However, to date, there are limited data regarding the impact of polymorphisms in drug-metabolizing enzymes and transporter genes on irinotecan-induced toxicity and efficacy in Thai colorectal cancer patients. This study aims to examine how these genetic variations affect irinotecan’s toxicity and efficacy in this population.

## Materials and methods

### Patient recruitment

**Fig 1:**
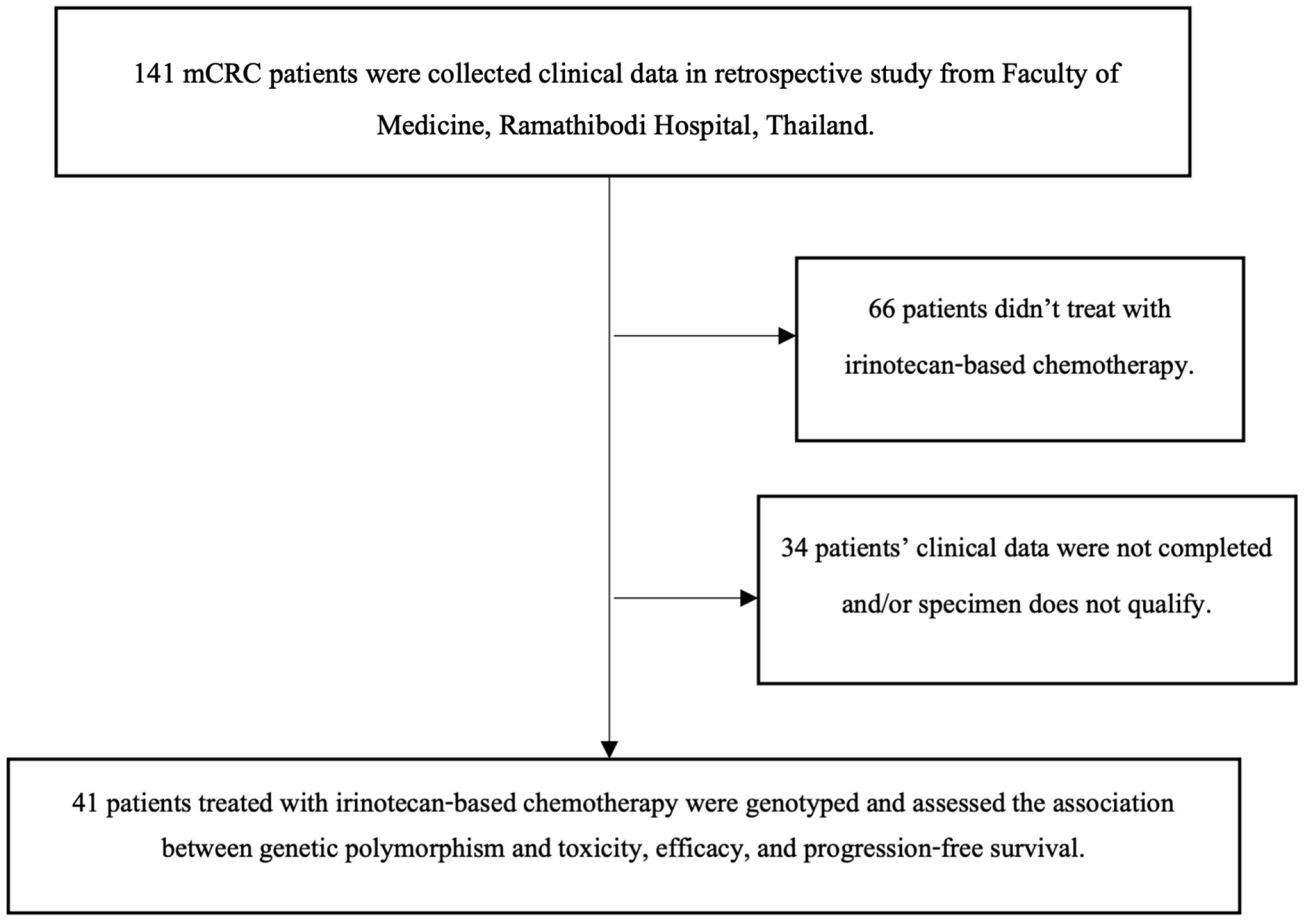
Flow chart of patients’ recruitment. The diagram illustrates the process of patient selection for the study. A total of 141 patients with colorectal cancer were initially screened. After applying the inclusion and exclusion criteria, 66 patients were found eligible, and 41 patients with complete clinical and genetic data were included in the final analysis.

A retrospective study recruited metastatic colorectal cancer patients who received irinotecan-based chemotherapy between August 2012 and June 2023 from the Division of Cancer, Department of Medicine, Faculty of Medicine, Ramathibodi Hospital, Mahidol University, Thailand. The inclusion criteria for patient enrollment were as follows: histologically confirmed invasive colorectal cancer, age > 18 years, ECOG status 0–2, no prior treatment with irinotecan, hemoglobin ≥13 g/dL, absolute neutrophil count (ANC) ≥1.5 × 10⁹/L, platelet count ≥8 × 10¹⁰/L, AST and ALT ≤ 2.5 times the upper normal limit (UNL), total bilirubin ≤2 mg/dL, and creatinine <2.0 mg/dL. The exclusion criteria included pregnancy, radiation therapy within six weeks, and genetic disorders related to colorectal cancer. A total of 41 metastatic colorectal cancer patients treated with irinotecan-based chemotherapy were assessed for the association between genetic polymorphisms and toxicity, efficacy, and progression-free survival. Fig 1 illustrates the patient screening flowchart (Fig 1). Clinical data were retrieved from electronic medical records in 2023 after obtaining ethical approval. This study used leftover human specimens and retrospective medical records. All data were fully anonymized prior to access and analysis, and no identifiable patient information was used. The Ethics Review Committee on Human Research, Faculty of Medicine, Ramathibodi Hospital, Mahidol University, Thailand (Reference No. MURA2023/472) reviewed and approved the protocol and waived the requirement for individual informed consent. The study was conducted in accordance with the Declaration of Helsinki.

### Treatment regimen

In this study, among 41 patients, eight received a single-agent irinotecan regimen consisting of irinotecan 100 mg/m2 administered as a 90-minute intravenous infusion on day 1. Eighteen patients received the standard FOLFIRI regimen, which included irinotecan 180 mg/m2 as a 90-minute intravenous infusion on day 1, leucovorin (LV) 200 mg/m2 as an intravenous infusion on day 1, fluorouracil 400 mg/m2 as an intravenous bolus on day 1, followed by fluorouracil 600 mg/m2 administered as a 46-hour continuous intravenous infusion. This regimen was repeated every two weeks. The remaining fifteen patients received a modified FOLFIRI regimen, consisting of irinotecan 180 mg/m2 as a 90-minute intravenous infusion on day 1, leucovorin (LV) 400 mg/m2 as an intravenous infusion on day 1, fluorouracil 400 mg/m2 as an intravenous bolus on day 1, followed by fluorouracil 1200 mg/m2 administered as a 46-hour continuous intravenous infusion, also repeated every two weeks.

### Genotyping analysis

Ethylenediaminetetraacetic acid (EDTA) whole blood was collected from enrolled patients and extracted DNA by using MagNA Pure Compact (MagNA Pure, Roche, Mannheim, Germany). DNA concentration and purity was measured with NanoDrop™ 2000 Spectrophotometer and adjusted the concentration to recommended for each genotyping platform by distilled water. Total 23 SNPs of UGT1A1*28, UGT1A1*6, CYP3A4*1B, CYP3A4*18, CYP3A5*3, *CES1* c.1165-33C > A, *CES1* c.1165-41G > A, *CES1* c.257 + 885A > G, *ABCB1* c.3435C > T, *ABCB1* c.1236C > T, *ABCB1* c. 2677C > A/T, *ABCG1* c. 286 + 7029C > T, *ABCG2* c.421C > A, *ABCG2* c. 34G > A, *ABCG2* c.1143C > T, *ABCG2* c.1738-46A > G, *ABCG2* c.1368-334C > T, *ABCG2* c.690-217A > G, *ABCC2* c.3972C > T, *ABCC2* c.-24C > T, *ABCC5* c.129 + 7980C > T, *SLCO1B1* c.521T > C, and *SLCO1B1* c.388A > G were performed by TaqMan Real-Time PCR (Genotype Applied Biosystems® ViiA™7 Real-Time PCR System, Applied Biosystems® ViiA™7, Carlsbad, CA, USA) and 2 SNPs of UGT1A1*6 (211G > A) and UGT1A1*28 ((TA)6>(TA)7) were performed by pyrosequencing (PyroMark Q24, Qiagen, Japan) analysis

### Outcome

The toxicity was assessed at the first and second cycle of treatment according to the National Cancer Institute Common Toxicity Criteria for Adverse Event (CTCAE) version 5.0. grades 1–2 were considered mild toxicity, and grades 3–4 as severe toxicity.

The efficacy of chemotherapy was evaluated according to the Response Evaluation Criteria in Solid Tumors (RECIST) version 1.1 [21], which classifies responses as complete response (CR), partial response (PR), stable disease (SD), or progressive disease (PD). Median progression-free survival (mPFS) was defined as the time from the first chemotherapy cycle to disease progression or death. Patients who were lost to follow-up, discontinued treatment, or switched to another chemotherapy regimen before documented disease progression were considered censored cases. The follow-up period for PFS was five years.

### Statistical analysis

Genetic polymorphisms were evaluated for adherence to Hardy–Weinberg equilibrium (HWE). The comparison of different groups of genetic polymorphisms, toxicity, and efficacy was performed using the *X*^*2*^ test and Fisher exact test. The data distribution was tested for normality, revealing a non-normal distribution. The Mann-Whitney U test assessed the comparison between different groups of genetic polymorphisms and nonparametric data [absolute neutrophil count (ANC)]. PFS curves were calculated using the Kaplan-Meier method and evaluated with the log-rank test. All statistical analyses were performed using SPSS version 28.0, with significance determined at *p* < 0.002 (following Bonferroni correction. The Bonferroni adjustment was applied by dividing the standard significance level (α = 0.05) by the number of tests performed in all analyses, resulting in the adjusted threshold to account for multiple testing and minimize type I error risk.

## Results

### Clinical characteristics

A total of 41 metastatic colorectal cancer patients who were receiving irinotecan-based chemotherapy were enrolled for analysis. Their clinical characteristics are shown in Table 1. The average age was 59.3 years (SD 9.4), with 73.2% males and 26.8% females. Most patients’ ECOG performance status was zero. The most common disease site was rectum, and the site of metastases was the liver and lung, The clinical characteristics of the patients are presented in Table 1.

**Table 1:** Clinical characteristics of patients with mCRC (n = 41).

Variable	Number	Percentage
Age (years)		
mean±SD	59.3 ± 9.4	
Gender		
Male	30	73.2
Female	11	26.8
ECOG score		
0	27	65.9
1	11	26.8
2	3	7.3
Tumor site		
Right side	3	7.3
Transverse colon	1	2.4
Left side	4	9.8
Sigmoid	10	24.4
Rectum	19	46.3
Rectosigmoid	4	9.8
More than two site	–	–
Sites of metastases		
No	2	4.9
Liver	14	34.1
Lung	5	12.2
Bone	1	2.4
Liver, Lung	16	39
Liver, Bone	3	7.3
Line of treatment		
First line	5	12.2
Second line	28	68.3
Third line	8	19.5
Treatment regimen		
Single irinotecan	8	19.5
FOLFIRI	18	43.9
Modified FOLFIRI	15	36.6

### Genotype and allele frequency

A total of 41 mCRC patients were genotyped for UGT1A1*28, UGT1A1*6, CYP3A4*1B, CYP3A4*18, CYP3A5*3, *ABCB1* c.3435C > T, *ABCB1* c.1236C > T, *ABCB1* c. 2677C > A/T, *ABCG1* c. 286 + 7029C > T, *ABCG2* c.421C > A, *ABCG2* c. 34G > A, *ABCG2* c.1143C > T, *ABCG2* c.1738-46A > G, *ABCG2* c.1368-334C > T, *ABCG2* c.690-217A > G, *ABCC2* c.3972C > T, *ABCC2* c.-24C > T, *ABCC5* c.129 + 7980C > T, *CES1* c.1165-33C > A, *CES1* c.1165-41G > A, and *CES1* c.257 + 885A > G, *SLCO1B1* c.521T > C, and *SLCO1B1* c.388A > G. The genotype and allele frequencies are shown in Table 2. The most prevalent variant alleles were *SLCO1B1* c.388A > G (72.0%), *ABCB1* c.2677C > T (72.0%), *ABCB1* c.1236C > T (63.4%), and CYP3A5*3 (57.3%), respectively. The variant of CYP3A4*1B was not detected.

**Table 2:** Genotype and allele frequency of drug-metabolizing enzyme and transporter genes in Thai colorectal cancer patients (n = 41).

Gene	Polymorphism	rs number	Genotype frequency n (%)			Allele frequency n (%)	
			Homozygous wild type	Heterozygous variant	Homozygous variant	Wild type allele	Variant allele
*UGT1A1*	*28 ((TA)7TAA)	rs 3064744	31 (75.6)	9 (22.0)	1 (2.4)	71 (87.0)	11 (13.0)
	*6 (211G > A)	rs4148323	34 (82.9)	7 (17.1)	0 (0.0)	75 (92.0)	7 (8.0)
*CYP3A4*	*1B (-392A > G)	rs2740574	41 (100.0)	0 (0.0)	0 (0.0)	82 (100.0)	0 (0.0)
	*18 (878T > C)	rs28371759	40 (97.6)	1 (2.4)	0 (0.0)	80 (97.6)	2 (2.4)
*CYP3A5*	*3 (6986A > G)	rs776746	6 (14.6)	23 (56.1)	12 (29.3)	35 (42.7)	47 (57.3)
*CES1*	1165-33C > A	rs2244613	17 (41.5)	14 (34.1)	10 (24.4)	48 (58.5)	34 (41.5)
	1165-41G > A	rs2244614	24 (58.5)	14 (34.1)	3 (7.3)	62 (75.6)	20 (24.4)
	257 + 885A > G	rs8192935	22 (53.7)	14 (34.1)	5 (12.2)	58 (70.7)	24 (29.3)
*ABCB1*	3435C > T	rs1045642	15 (36.6)	20 (48.8)	6 (14.6)	50 (61.0)	32 (39.0)
	1236C > T	rs1128503	5 (12.2)	20 (48.8)	16 (39.0)	30 (36.6)	52 (63.4)
	2677C > A	rs2032582	10 (24.4)	19 (46.3)	12 (29.3)	39 (47.6)	43 (52.4)
	2677C > T	rs2032582	10 (24.40	3 (7.3)	28 (68.3)	23 (28.0)	59 (72.0)
*ABCG1*	286 + 7029C > T	rs225440	20 (48.8)	18 (43.9)	3 (7.3)	58 (70.7)	24 (29.3)
*ABCG2*	421C > A	rs2231142	25 (61.0)	15 (36.6)	1 (2.4)	65 (79.3)	17 (20.7)
	34G > A	rs2231137	13 (31.7)	21 (51.2)	7 (17.1)	47 (57.3)	35 (42.7)
	1143C > T	rs2622604	25 (61.0)	15 (36.6)	1 (2.4)	65 (79.3)	17 (20.7)
	1738-46A > G	rs2231164	11 (26.8)	17 (41.5)	13 (31. 7)	39 (47.6)	43(52.4)
	1368-334C > T	rs4148157	22 (53.7)	13 (31.7)	6 (14.6)	57 (69.5)	25 (30.5)
	690-217A > G	rs1871744	18 (43.9)	17 (41.5)	6 (14.6)	53 (64.6)	29 (35.4)
*ABCC2*	3972C > T	rs3740066	24 (58.5)	16 (39.0)	1 (2.4)	64 (78.0)	18 (22.0)
	-24C > T	rs717620	27 (65.9)	10 (24.4)	4 (9.8)	64 (78.0)	18 (22.0)
*ABCC5*	129 + 7980C > T	rs2292997	20 (48.8)	17 (41.5)	4 (9.8)	57 (69.5)	25 (30.5)
*SLCO1B1*	521T > C	rs4149056	33 (80.5)	8 (19.5)	0 (0.0)	66 (80.5)	16 (19.5)
	388A > G	rs2306283	7 (17.1)	16 (39.0)	18 (43.9)	23 (28.0)	59 (72.0)

**Fig 2:**
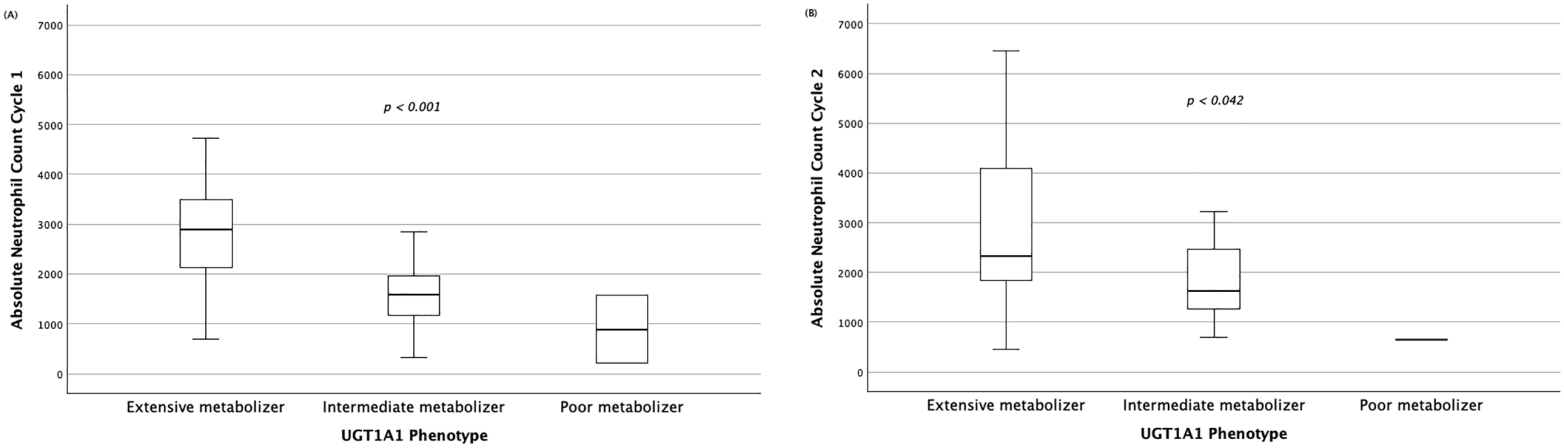
Association of UGT1A1 phenotype with absolute neutrophil count during irinotecan-based chemotherapy. Box plots show the distribution of absolute neutrophil counts across UGT1A1 phenotypes (extensive, intermediate, and poor metabolizers) in the (A) first cycle and (B) second cycle of treatment. Statistical comparisons were performed using the Kruskal–Wallis test.

The association between drug-metabolizing gene polymorphisms and irinotecan-induced neutropenia is summarized in Table 3. The UGT1A1*6 was significantly associated with both all-grade and severe neutropenia in the first cycle (*p *< 0.001) and severe neutropenia in the second cycle (*p* < 0.002). The UGT1A1 phenotype, including intermediate and poor metabolizers, was linked to a higher incidence of all-grade neutropenia during the first cycle (*p *< 0.001) and severe neutropenia during the second cycle (*p* < 0.001). In the same direction as the results observed under the dominant model (S1 and S2 Tables). Additionally, intermediate and poor metabolizers exhibited lower absolute neutrophil count nadirs than extensive metabolizers in the first cycle (*p* < 0.001) (Fig 2A) and second cycle (*p* = 0.042) (Fig 2B).

**Table 3:** Impacts of polymorphisms in drug-metabolizing enzyme genes on irinotecan-induced neutropenia in the first and second cycle (n = 41).

Gene	Allele	n	Toxicity (neutropenia)							
			First Cycle				Second Cycle			
			Grade 1–4	*p*	Grade 3–4	*p*	Grade 1–4	*p*	Grade 3–4	*p*
			n (%)		n (%)		n (%)		n (%)	
*UGT1A1*										
*28 (TA)7TAA)	TA6	71	28 (39.4)	0.008	9 (12.7)	1	31 (43.7)	0.143	13 (18.3)	0.375
	TA7	11	8 (72.7)		1 (9.1)		7 (63.6)		3 (27.3)	
*6 (211G > A)	G	75	29 (38.7)	<0.001*	6 (8.0)	<0.001*	32 (42.7)	0.003	12 (16.0)	<0.002*
	A	7	7 (100.0)		4 (57.1)		6 (85.7)		4 (57.1)	
UGT1A1 phenotype										
*1/*1	Extensive metabolizer	25	5 (20.0)	<0.001*	1 (4.0)	0.018	8 (32.0)	0.004	3 (12.0)	<0.001*
*1/*28, *1/*6	Intermediate metabolizer	14	11 (78.6)		3 (21.4)		9 (64.3)		3 (21.4)	
*28/*28, *28/*6	Poor metabolizer	2	2 (100.0)		1 (50.0)		2 (100.0)		2 (100.0)	
*CYP3A4*										
*1B (c.-392A > G)	A	82	36 (43.9)	NA	10 (12.2)	NA	38 (46.3)	NA	16 (19.5)	NA
*18 (c.878T > C)	T	80	36 (45.0)	0.131	10 (12.5)	1.000	38 (47.5)	0.123	16 (20.0)	1.000
	C	2	0 (0.0)		0 (0.0)		0 (0.0)		0 (0.0)	
*CYP3A5*										
*3 (c.6986A > G)	A	35	16 (45.7)	0.747	3 (8.6)	0.558	18 (51.4)	0.368	6 (17.1)	0.603
	G	47	20 (42.6)		7 (14.9)		20 (42.6)		10 (21.3)	
*CES1*										
rs2244613 (c.1165-33C > A)	C	48	20 (41.7)	0.565	6 (12.5)	1.000	20 (41.7)	0.231	8 (16.7)	0.369
	A	34	16 (47.1)		4 (11.8)		18 (52.9)		8 (23.5)	
rs2244614 (c.1165-41G > A)	G	62	28 (45.2)	0.607	8 (12.9)	1.000	28 (45.2)	0.633	11 (17.7)	0.376
	A	20	8 (40.0)		2 (10.0)		10 (50.0)		5 (25.0)	
rs8192935 (c.257 + 885A > G)	A	58	27 (46.6)	0.348	8 (13.8)	0.540	27 (46.6)	0.941	11 (19.0)	1.000
	G	24	9 (37.5)		2 (8.3)		11 (45.8)		5 (20.8)	

Note. Genetic polymorphism associated with neutropenia in first and second cycle of irinotecan-base regimen treatment in 41 mCRC patients. N/A does not analyze, value with * indicate the statistically significant with Bonferroni-corrected (*p* value < 0.002), grades 1–4 was considered as toxicity and grades 3–4 was considered as severe toxicity.

For drug transporter genes, the results are described in Table 4. The *ABCC2* c.-24C > T polymorphism was significantly associated with all-grade neutropenia in the second cycle (*p* = 0.001). However, other polymorphisms did not show statistically significant associations with neutropenia toxicity after applying the Bonferroni correction.

**Table 4:** Impacts of polymorphisms in drug transporter genes on irinotecan-induced neutropenia in first and second cycle (n = 41).

Gene	Allele	n	Toxicity (neutropenia)							
			First Cycle				Second Cycle			
			Grade 1–4	*p*	Grade 3–4	*p*	Grade 1–4	*p*	Grade 3–4	*p*
			n (%)		n (%)		n (%)		n (%)	
*ABCB1*										
rs1045642 (c.3435T > C)	C	50	20 (40.0)	0.287	6 (12.0)	1.000	21 (42.0)	0.238	8 (16.0)	0.242
	T	32	16 (50.0)		4 (12.5)		17 (53.1)		8 (25.0)	
rs1128503 (c.1236C > T)	C	30	12 (40.0)	0.550	5 (16.7)	0.281	13 (43.3)	0.647	5 (16.7)	0.589
	T	52	24 (46.2)		5 (9.6)		25 (48.1)		11 (21.2)	
rs2032582 (c.2677C > A)	C	39	17 (43.6)	0.950	5 (12.8)	0.849	19 (48.7)	0.637	9 (23.1)	0.363
	A	43	19 (44.2)		5 (11.6)		19 (44.2)		7 (16.3)	
rs2032582 (c.2677C > T)	C	23	8 (34.8)	0.264	2 (8.7)	0.738	7 (30.4)	0.052	2 (8.7)	0.162
	T	59	28 (47.5)		8 (13.6)		31 (52.5)		14 (23.7)	
*ABCG1*										
rs225440 (c.286 + 7029C > T)	C	58	26 (44.8)	0.744	7 (12.1)	1.000	26 (44.8)	0.595	12 (20.7)	0.628
	T	24	10 (41.7)		3 (12.5)		12 (50.0)		4 (16.7)	
*ABCG2*										
rs2231142 (c.421C > A)	C	65	30 (46.2)	0.299	8 (12.3)	1.000	30 (46.2)	0.932	14 (21.5)	0.282
	A	17	6 (35.3)		2 (11.8)		8 (47.1)		2 (11.8)	
rs2231137 (c.34G > A)	G	47	19 (40.4)	0.386	7 (14.9)	0.371	22 (46.8)	0.907	9 (19.1)	0.910
	A	35	17 (48.6)		3 (8.6)		16 (45.7)		7 (20.0)	
rs2622604 (c.1143C > T)	G	65	29 (44.6)	0.743	10 (15.4)	0.014	32 (49.2)	0.185	15 (23.1)	0.047
	A	17	7 (41.2)		0 (0.0)		6 (35.3)		1 (5.9)	
rs2231164 (1738-46A > G)	A	39	19 (48.7)	0.336	4 (10.3)	0.774	17 (43.6)	0.586	7 (17.9)	0.699
	G	43	17 (39.5)		6 (14.0)		21 (48.8)		9 (20.9)	
rs4148157 (c.1368-334C > T)	G	57	28 (49.1)	0.073	6 (10.5)	0.567	29 (50.9)	0.122	13 (22.8)	0.205
	A	25	8 (32.0)		4 (16.0)		9 (36.0)		3 (12.0)	
rs1871744 (c.690-217A > G)	A	53	19 (35.8)	0.016	7 (13.2)	0.770	22 (41.5)	0.150	8 (15.1)	0.110
	G	29	17 (58.6)		3 (10.3)		16 (55.2)		8 (27.6)	
*ABCC2*										
rs3740066 (c.3927C > T)	C	64	25 (39.1)	0.034	9 (14.1)	0.319	26 (40.6)	0.012	11 (17.2)	0.212
	T	18	11 (61.1)		1 (5.6)		12 (66.7)		5 (27.8)	
rs717620 (c.-24C > T)	C	64	25 (39.1)	0.034	9 (14.1)	0.319	25 (39.1)	0.001*	12 (18.8)	0.795
	T	18	11 (61.1)		1 (5.6)		13 (72.2)		4 (22.2)	
*ABCC5*										
rs2292997 (c.129 + 7980C > T)	C	57	28 (49.1)	0.073	7 (12.3)	1.000	29 (50.9)	0.122	10 (17.5)	0.410
	T	25	8 (32.0)		3 (12.0)		9 (36.0)		6 (24.0)	
*SLCO1B1*										
*5 (c.521T > C)	T	66	30 (45.5)	0.456	6 (9.1)	0.061	30 (45.5)	0.672	12 (18.2)	0.436
	C	16	6 (37.5)		4 (25.0)		8 (50.0)		4 (25.0)	
*1b (c.388A > G)	A	23	11 (47.8)	0.629	1 (4.3)	0.313	12 (52.2)	0.475	5 (21.7)	0.730
	G	59	25 (42.4)		9 (15.3)		26 (44.1)		11 (18.6)	
OATP1B1 function										
*1/*1, *1b/*1b	Normal function	33	15 (45.5)	0.596	3 (9.1)	0.146	15 (45.5)	0.792	6 (18.2)	0.743
*5/*1b	Decrease function	0	0 (0.0)		0 (0.0)		0 (0.0)		0 (0.0)	
*5/*5	Poor function	8	3 (37.5)		2 (25.0)		4 (50.0)		2 (25.0)	

Note. Genetic polymorphism associated with neutropenia in first and second cycle of irinotecan-base regimen treatment in 41 mCRC patients. N/A does not analyze, value with * indicate the statistically significant with Bonferroni-corrected (P value < 0.002), grades 1–4 was considered as toxicity and grades 3–4 was considered as severe toxicity.

### Impacts of polymorphisms in drug-metabolizing enzyme and transporter genes on treatment efficacy

In this study, we evaluated efficacy outcomes by assessing response rates and progression-free survival (PFS). There was no significant association in response rates observed among the 21 SNP variants analyzed. The summarized results of genetic polymorphisms in drug-metabolizing enzymes and transporter genes affecting response rates are presented in Tables 5 and 6, respectively. In the same direction as the results observed under the dominant model (S3 and S4 Tables).

**Table 5:** Impacts of polymorphisms in drug-metabolizing enzyme genes on response rates (n = 41).

Gene	Genotype	n	Response Rates		
			Non-responder	Responder	*p*
			(SD + PD)	(CR + PR)	
*UGT1A1*					
*28 ((TA)7TAA)	TA6	71	67 (94.4)	4 (5.6)	0.569
	TA7	11	11 (100.0)	0 (0.0)	
*6 (211G > A)	G	75	71 (94.7)	4 (5.3)	1.000
	A	7	7 (100.0)	0 (0.0)	
UGT1A1 phenotype					
*1/*1	Extensive metabolizer	25	23 (92.0)	2 (8.0)	0.245
*1/*6, *1/*28	Intermediate metabolizer	14	14 (100.0)	0 (0.0)	
*28/*6, *28/*28	Poor metabolizer	2	2 (100.0)	0 (0.0)	
*CYP3A4*					
*1B (c.-392A > G)	A	82	78 (95.1)	4(4.9)	NA
*18 (c.878T > C)	T	80	76 (95.0)	4 (5.0)	1.000
	C	2	2 (100.0)	0 (0.0)	
*CYP3A5*					
*3 (c.6986A > G)	A	35	34 (97.1)	1 (2.9)	0.673
	G	47	44 (93.6)	3 (6.4)	
*CES1*					
rs2244613 (c.1165-33C > A)	C	48	46 (85.8)	2 (4.2)	1.000
	A	34	32 (94.1)	2 (5.9)	
rs2244614 (c.1165-41G > A)	G	62	60 (96.8)	2 (3.2)	0.207
	A	20	18 (90.0)	2 (10.0)	
rs8192935 (c.257 + 885A > G)	A	58	56 (96.6)	2 (3.4)	0.407
	G	24	22 (91.7)	2 (8.3)	

Note. Genetic polymorphism is associated with the efficacy of irinotecan-based regimen treatment in 41 mCRC patients. N/A does not analyze, value with * indicate the statistically significant with Bonferroni-corrected (*p* < 0.002), non-responder was considered for stable disease (SD) and progressive (PD) disease and responder was considered for complete response (CR) and partial response (PR).

**Table 6:** Impacts of polymorphisms in drug transporter genes on response rates (n = 41).

Gene	Genotype	n	Response Rates		
			Non-responder	Responder	*p*
			(SD + PD)	(CR + PR)	
*ABCB1*					
rs1045642 (c.3435T > C)	T	50	48 (96.0)	2 (4.0)	0.694
	C	32	30 (93.8)	2 (6.3)	
rs1128503 (c.1236C > T)	C	30	29 (96.7)	1 (3.3)	1.000
	T	52	49 (94.2)	3 (5.8)	
rs2032582 (c.2677C > A)	C	39	36 (92.3)	3 (7.7)	0.175
	A	43	42 (97.7)	1 (2.3)	
rs2032582 (c.2677C > T)	C	23	21 (91.3)	2 (8.7)	0.253
	T	59	57 (96.6)	2 (3.4)	
*ABCG1*					
rs225440 (c.286 + 7029C > T)	C	58	56 (96.6)	2 (3.4)	0.407
	T	24	22 (91.7)	2 (8.3)	
*ABCG2*					
rs2231142 (c.421C > A)	C	65	61 (93.8)	4 (6.2)	0.296
	A	17	17 (100.0)	0 (0.0)	
rs2231137 (c.34G > A)	G	47	44 (93.6)	3 (6.4)	0.390
	A	35	34 (97.1)	1 (2.9)	
rs2622604 (c.1143C > T)	C	65	61 (93.8)	4 (6.2)	0.296
	T	17	17 (100.0)	0 (0.0)	
rs2231164 (1738-46A > G)	A	39	38 (97.4)	1 (2.6)	0.433
	G	43	40 (93.0)	3 (7.0)	
rs4148157 (c.1368-334C > T)	C	57	53 (93.0)	4 (7.0)	0.121
	T	25	25 (100.0)	0 (0.0)	
rs1871744 (c.690-217A > G)	A	53	52 (98.1)	1 (1.9)	0.116
	G	29	26 (89.7)	3 (10.3)	
*ABCC2*					
rs3740066 (c.3927C > T)	C	64	61 (95.3)	3 (4.7)	1.000
	T	18	17(94.4)	1 (5.6)	
rs717620 (c.-24C > T)	C	64	60 (93.8)	4 (6.3)	0.294
	T	18	18 (100.0)	0 (0.0)	
*ABCC5*					
rs2292997 (c.129 + 7980C > T)	C	57	55 (96.5)	2 (3.5)	0.415
	T	25	23 (92.0)	2 (8.0)	
*SLCO1B1*					
rs4149056 (c.521T > C)	T	66	62 (93.9)	4 (6.1)	0.300
	C	16	16 (100.0)	0 (0.0)	
rs2306283 (c.388A > G)	A	23	21 (91.3)	2 (8.7)	0.253
	G	59	57 (96.6)	2 (3.4)	
OATP1B1 function					
*1/*1, *1b/*1b	Normal function	33	31 (93.9)	2 (6.1)	0.504
*5/*1b	Decrease function	0	0 (0.0)	0 (0.0)	
*5/*5	Poor function	8	8 (100.0)	0 (0.0)	

Note. Genetic polymorphism is associated with the efficacy of irinotecan-based regimen treatment in 41 mCRC patients. N/A does not analyze, value with * indicate the statistically significant with Bonferroni-corrected (*p* < 0.002), non-responder was considered for stable disease (SD) and progressive (PD) disease and responder was considered for complete response (CR) and partial response (PR).

**Fig 3:**
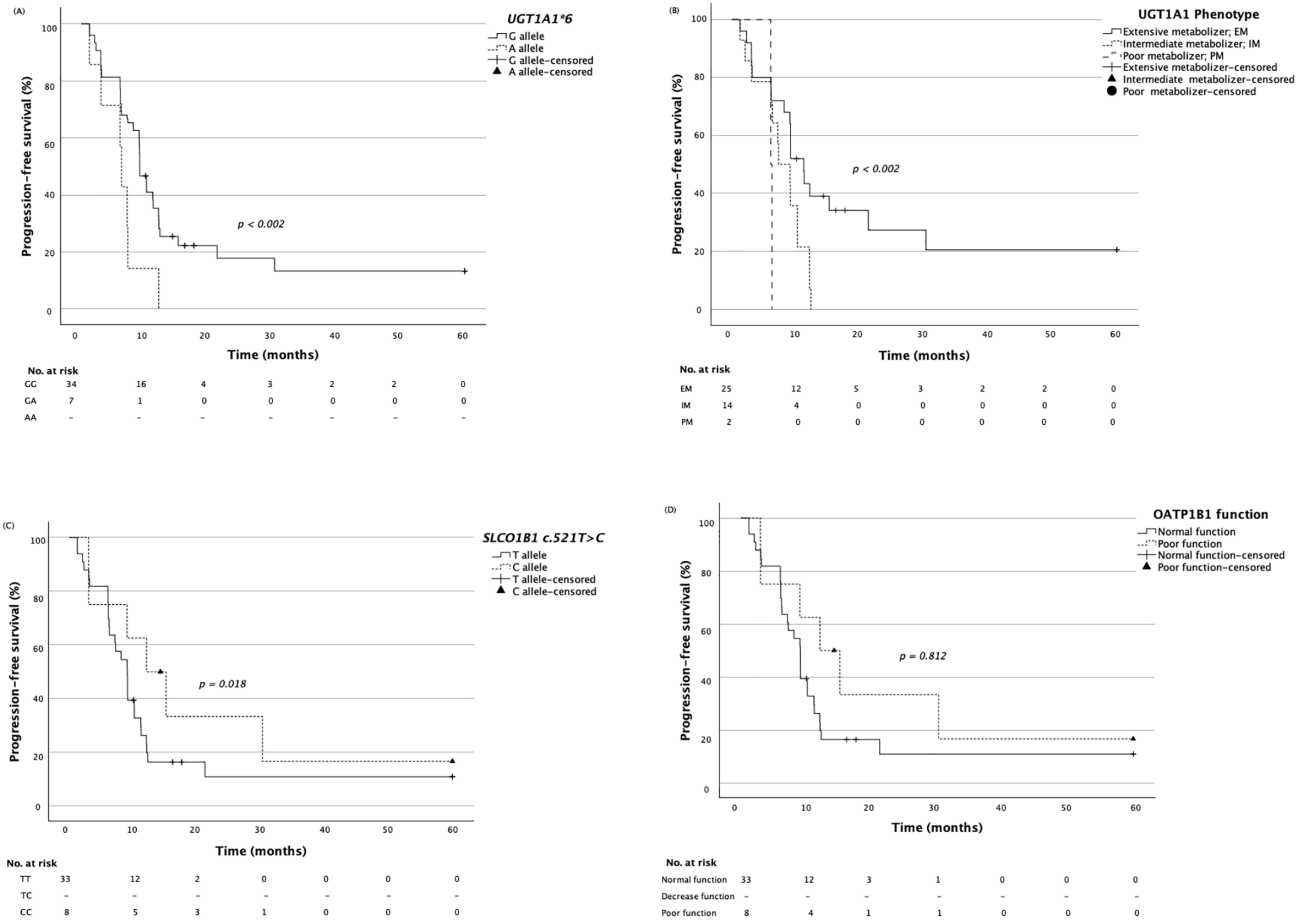
Kaplan–Meier curves of progression-free survival (PFS) stratified by (A) UGT1A1*6, (B) UGT1A1 phenotype, (C) *SLCO1B1* c.521C > T, and (D) OATP1B1 function. Median progression-free survival (mPFS) was defined as the time from the first chemotherapy cycle to disease progression or death. Patients who were lost to follow-up, discontinued treatment, or switched to another chemotherapy regimen before documented disease progression were considered censored cases. The follow-up period for PFS was five years. The numbers of patients at risk at each time point are shown below the curves, and censored observations are indicated by ‘+’, ‘▲’, or ‘●’. Survival was estimated using the Kaplan–Meier method and compared using the log-rank test.

In progression-free survival assessment, regarding drug-metabolizing genes, the median survival time for all 41 patients was 9.11 months (range: 6.1–12.1). Patients with the UGT1A1*6 variant exhibited significantly shorter PFS compared to those with the wild type (6.2 vs 12.1 months, 95% CI = 5.8–6.7, *p* < 0.002) (Fig 3A). In intermediate and poor metabolizers, PFS is shorter compared to extensive metabolizers, but this difference is not significant after applying Bonferroni correction (7.7 vs 13.4 months, 95% CI = 4.8–9.7, *p* = 0.006 for intermediate metabolizer and 6.1 vs 13.4 months, 95% CI = 6.7–13.4, *p* = 0.009 for poor metabolizer) (Fig 3B).

Conversely, the PFS for patients carrying the *SLCO1B1* c.521T > C variant was significantly longer than that of wild type patients (17.1 vs 9.6 months, 95% CI = 7.9–16.3, *p* < 0.002) (Fig 3C). However, progression-free survival (PFS) for poor-function OATP1B1 seem longer than for normal function, though the difference is not statistically significant (11.0 vs 9.6 months, 95% CI = 9.0–9.2, *p* = 0.812) (Fig 3D). No statistically significant associations were found between other polymorphisms and PFS.

## Discussion

This study demonstrated the impact of genetic polymorphisms in drug-metabolizing enzymes and drug transporter genes on irinotecan-induced neutropenia and treatment efficacy in Thai patients with colorectal cancer. Statistical significance was achieved in this study using the Bonferroni correction, enhancing the reliability and robustness of the research findings. Applying the stringent Bonferroni correction (*p* value < 0.002), our findings demonstrated a significant association between irinotecan-induced neutropenia and the UGT1A1*6 and UGT1A1 phenotypes, as well as the *ABCC2* c.-24C > T variant, in relation to toxicity. In addition, the UGT1A1*6 and *SLCO1B1* c.521T > C variants showed a strong association with progression-free survival. However, other drug-metabolizing enzymes and drug transporter genes did not show significantly associated with irinotecan-induced toxicity and treatment efficacy.

UGT1A1 is currently known as the main isozyme involved in irinotecan metabolism [5]. Variations in the *UGT1A1* gene, particularly UGT1A1*28 and UGT1A1*6, are associated with decreased UGT1A1 activity [8], leading to the accumulation of SN-38 and an increased risk of adverse reactions and therapeutic effects in patients with advanced CRC undergoing irinotecan-based chemotherapy [10,22]. Numerous studies have examined the relationship between the UGT1A1*28 polymorphism and irinotecan-induced toxicity [23,24]. A study by Rouits et al. found that individuals with TA6/TA7 and TA7/TA7 genotypes exhibited an increased risk of neutropenia compared to those with the UGT1A1*1 genotype [25]. However, a recent study from Guangxi Zhuang in China also found that UGT1A1*28 was not associated with irinotecan-related neutropenia in patients with metastatic colorectal cancer [26]. Our study found that the incidence of all-grade neutropenia during the first cycle was higher in patients with the TA7 allele (UGT1A1*28) compared to those with the TA6 allele (*p* = 0.008). Nonetheless, after applying the Bonferroni correction, the association between UGT1A1*28 and neutropenia was no longer statistically significant. In contrast, UGT1A1*6 remained significantly associated with an increased risk of all-grade neutropenia in the first cycle and severe neutropenia in both cycles (*p* < 0.002). Our finding is consistent with the study by Atasilp et al. in a Thai population, which found that UGT1A1*6 was significantly associated with both all-grade and severe neutropenia in Thai patients. While no significant association was observed between UGT1A1*28 and severe neutropenia [9].

Additionally, when considering the UGT1A1 phenotype based on a combined analysis of UGT1A1*6 and UGT1A1*28, individuals with two normal function alleles (*1/*1) are classified as extensive metabolizers. Those carrying one normal function allele (*1) and one reduced function allele (*6 or *28) are classified as intermediate metabolizers (IMs). Meanwhile, individuals with two reduced function alleles (such as *28/*28 or *6/*6) are classified as poor metabolizers (PMs) [27]. Our study demonstrated that intermediate and poor metabolizers of UGT1A1 were significantly associated with both all-grade and severe neutropenia in the first and second cycles. Moreover, after applying the Bonferroni correction, the UGT1A1 phenotype remained significantly associated with all-grade neutropenia in the first cycle and severe neutropenia in the second cycle.

Since the prevalence of the UGT1A1*28 variant is relatively high among Caucasians and Africans/African Americans [28,29], it is significantly lower in the Asian population [28,29]. In contrast, the UGT1A1*6 polymorphism is more common in Asian populations [30,31]. Given its higher prevalence in Asian populations, the UGT1A1*6 polymorphism may serve as a better predictor of irinotecan-related neutropenia in the Thai population. Nevertheless, a combined analysis of UGT1A1*6 and UGT1A1*28 may also serve as a potential biomarker for irinotecan-induced neutropenia in the Thai population, which still requires validation through further research. Beyond Thailand, *UGT1A1* testing has been recognized internationally as a clinically relevant biomarker for irinotecan safety. The U.S. Food and Drug Administration (FDA) label for irinotecan includes a warning for patients carrying the UGT1A1 *28/*28 genotype due to their increased risk of severe neutropenia. Similarly, the European Medicines Agency (EMA) and the Japanese Pharmaceuticals and Medical Devices Agency (PMDA) acknowledge the utility of *UGT1A1* genotyping in guiding irinotecan dosing. Several clinical guidelines recommend considering *UGT1A1* testing prior to irinotecan initiation, particularly in populations with higher prevalence of risk alleles.

In terms of the efficacy of irinotecan-based chemotherapy, our study revealed that treatment response was not significantly associated with either the UGT1A1*28 or UGT1A1*6 gene polymorphisms. Previous observational studies examining the impact of UGT1A1*6 or *28 on clinical efficacy have produced inconsistent results. A previous meta-analysis suggested a trend towards higher response rates in patients with the UGT1A1*28 (TA)7 allele [32]. In contrast, another study reported that the UGT1A1*28 variant genotype was associated with worse progression-free survival and overall survival compared to the wild type genotype [33]. Other reviews indicate that the UGT1A1*28 polymorphism was not linked to any alterations in the objective response rate, progression-free survival, or overall survival after irinotecan treatment [34,35]. A recent study also revealed that neither UGT1A1*6 nor UGT1A1*28 affected treatment efficacy or progression-free survival in metastatic colorectal cancer [26]. Similarly, the study by Li et al. found no significant differences in response rate or PFS among different genotypes [36]. In addition, Han et al. [37] demonstrated that Korean patients with non-small-cell lung cancer who had the UGT1A1*6/*6 genotype and were treated with irinotecan and cisplatin experienced reduced tumor response rates, along with shorter progression-free survival and overall survival, compared to patients with other genotypes. Aligning with our study, we found that patients carrying the UGT1A1*6 variant had a significantly shorter progression-free survival compared to those with the wild type of genotype (*p* < 0.002). The UGT1A1*6 variant might be linked to poorer progression-free survival; however, the UGT1A1 phenotype showed no significant association with progression-free survival after Bonferroni correction.

There is no consensus on whether the polymorphism of the *UGT1A1* gene can predict the efficacy of irinotecan. The lack of differences in chemotherapy outcomes among the various genotypes may be due to the retrospective nature of this study, the small sample size, and the non-uniform dosage of irinotecan. Therefore, future studies should consider not only the *UGT1A1* gene polymorphism but also factors such as patients’ status, the intensity of drug dosage, the duration of treatment, the combination of medications, and other relevant elements when predicting clinical outcomes to confirm the impact of *UGT1A1* gene polymorphisms on the clinical efficacy of irinotecan-based chemotherapy.

For other drug-metabolizing genes, including *CYP3A4, CYP3A5,* and *CES1,* no significant associations with irinotecan-induced toxicity or clinical efficacy were observed. These findings may reflect the limited sample size, underscoring the need for larger cohorts to more accurately evaluate the impact of these genes on irinotecan response and toxicity.

Regarding drug-transporter genes, ATP-binding cassette (ABC) transporters, which are efflux transporters, play a crucial role in the pharmacokinetics of irinotecan and its active metabolite, SN-38. Key ABC transporters involved in the disposition of irinotecan and SN-38 include *ABCB1*, *ABCC2*, *ABCC5*, *ABCG1*, and *ABCG2* [7,38]. After Bonferroni correction, our study observed that only the *ABCC2* c.-24C > T (rs717620) variant was significantly associated with all-grade neutropenia in the second cycle. The *ABCC2 c.-24T* variant has been associated with lower mRNA levels, reduced activity in renal tissues, and decreased activity in a reporter gene assay [39]. This suggests that patients with the *ABCC2* c.-24T variant may experience overexposure to SN-38, likely due to decreased biliary clearance. This is consistent with the findings of Akiyama Y and colleagues, who reported that the *ABCC2* c.-24C > T polymorphism was significantly associated with increased SN-38 exposure, as measured by the area under the time-concentration curve [40]. Additionally, a haplotype in the multidrug transporter *ABCC2* has been linked to toxicity in patients without the UGT1A1*28 variant [16,41].

Concerning clinical efficacy, previous studies have shown that patients with the *ABCC2* c.-24T homozygous genotype had significantly better response rates and progression-free survival in non-small-cell lung cancer patients treated with irinotecan and cisplatin [42]. Metastatic colorectal cancer patients without the *ABCC2* c.-24T variant showed an increased overall response rate and median progression-free survival (*p* = 0.031) [43]. However, several published studies have reported conflicting data. Some studies have explored the relationship between *ABCC2* polymorphisms and chemotherapy response rates in Asians, finding that patients with the C/C genotype at −24 in *ABCC2* had a higher overall response rate compared to those with the C/T or T/T genotypes [43]. In contrast, Treenert and colleagues reported that the *ABCC2* c.-24C > T variant was not significantly associated with treatment responses [44], which is consistent with our findings that showed no statistically significant differences in response rates or progression-free survival among the *ABCC2* c.-24C > T variants.

Nevertheless, no statistically significant associations were observed for other efflux transporter genes with either clinical efficacy or toxicity. This may be due to the limited sample size and follow-up duration, highlighting the need for larger, long-term prospective studies to clarify the role of these genes in treatment outcomes.

In the field of influx transporters, SLCO1B1 plays a key role in mediating the hepatic uptake of SN38 from the bloodstream. Numerous clinical studies have evaluated the impact of *SLCO1B1* variants in irinotecan treatment. One study reported that the rs2306283 variant of *SLCO1B1* was associated with a higher rapid response rate, longer progression-free survival, and irinotecan-related time to treatment failure in mCRC patients treated with FOLFIRI/mCapeIRI regimens, while the rs4149056 variant did not predict rapid response rate or survival [45]. However, another study found no statistically significant association between the rs2306283 variant of *SLCO1B1* and AUCSN38 or tumor response in lung cancer patients [46]. The *SLCO1B1* c.388A > G polymorphism may have minimal impact on SLCO1B1 activity, its effect on drug pharmacokinetics remains under investigation [47]. This is consistent with our findings, as no statistically significant associations were observed between rs2306283 (*SLCO1B1* c.388A > G) and either toxicity or efficacy. Notably, however, the *SLCO1B1* c.521T > C polymorphism remained significantly associated with progression-free survival after correction, with carriers of the C allele demonstrating longer progression-free survival compared with those harboring the T allele.The C allele of rs4149056 has been linked to decreased SLCO1B1 membrane expression, reduced transport activity, lower drug clearance [48,49], and higher plasma AUCSN38 levels [46,47]. Furthermore, a prospective study examined the impact of the functional *SLCO1B1* c.521T > C variant on plasma SN-38 levels at the conclusion of irinotecan infusion, showing that patients with the *521T > C* variant had higher plasma concentrations of SN-38 compared to those without the variant [20]. It is possible that patients with the *SLCO1B1 C* allele had higher plasma levels of SN-38, potentially contributing to their longer progression-free survival. However, in our study, the *SLCO1B1* c.521T > C variant was not significantly associated with irinotecan-induced neutropenia, which may be due to the small sample size. Additionally, some studies have found no significant association between this variant and SN-38 pharmacokinetics [19,50]. To clarify these findings, larger, well-powered studies are warranted to further investigate the relationships among *SLCO1B1* variants, SN-38 plasma levels, and treatment outcomes.

This study has some limitations. Its retrospective design required the exclusion of numerous samples with incomplete data, resulting in a relatively small cohort. Consequently, the limited sample size precluded multivariable analyses and constrained statistical power, as well as the assessment of allele frequencies. Larger, prospective studies are needed to clarify the impact of drug-metabolizing enzyme and transporter genes on irinotecan toxicity and efficacy in Thai patients with colorectal cancer.

## Conclusions

In conclusion, UGT1A1*6 and *ABCC2* -24C > T *p*olymorphisms may serve as predictors of irinotecan-induced neutropenia, while UGT1A1*6 and *SLCO1B1* 521T > C are associated with improved progression-free survival in Thai patients. These findings support the incorporation of pharmacogenetic testing into clinical practice to optimize irinotecan dosing, minimize toxicity, and improve therapeutic outcomes in colorectal cancer. Future prospective studies with larger cohorts are needed to validate and extend these findings.

## Supporting information

S1 Table Impacts of polymorphisms in drug-metabolizing enzyme genes on irinotecan-induced neutropenia in the first and second cycle (Dominant Model). This table summarizes the associations between genetic polymorphisms in drug-metabolizing enzyme genes and irinotecan-induced neutropenia during the first and second cycles of irinotecan-based treatment in 41 patients with mCRC. Analyses were conducted using the dominant genetic model, comparing individuals carrying at least one variant allele with those homozygous for the wild-type allele.
(DOCX)

S2 Table Impacts of polymorphisms in drug-transporter genes on irinotecan-induced neutropenia in the first and second cycle (Dominant Model). This table summarizes the associations between genetic polymorphisms in drug-transporter genes and irinotecan-induced neutropenia during the first and second cycles of irinotecan-based treatment in 41 patients with mCRC. Analyses were conducted using the dominant genetic model, comparing individuals carrying at least one variant allele with those homozygous for the wild-type allele.
(DOCX)

S3 Table Impacts of polymorphisms in drug-metabolizing enzyme genes on response rates (Dominant Model). This table summarizes the associations between genetic polymorphisms in drug-metabolizing enzyme genes and treatment response rates in 41 patients receiving irinotecan-based therapy for mCRC. Analyses were conducted using the dominant genetic model, comparing individuals carrying at least one variant allele with those homozygous for the wild-type allele.
(DOCX)

S4 Table Impacts of polymorphisms in drug transporter genes on response rates. **(Dominant Model).** This table summarizes the associations between genetic polymorphisms in drug-transporter genes and treatment response rates in 41 patients receiving irinotecan-based therapy for mCRC. Analyses were conducted using the dominant genetic model, comparing individuals carrying at least one variant allele with those homozygous for the wild-type allele.
(DOCX)
