# Supplementary material for: Impacts of polymorphisms in drug-metabolizing enzyme and transporter genes on irinotecan toxicity and efficacy in Thai colorectal cancer patients
Source: PLoS One. 2025 Dec 12;20(12):e0338442. doi: 10.1371/journal.pone.0338442 (PMC12700395; doi:10.1371/journal.pone.0338442)
Supplement: S1 Table — This table summarizes the associations between genetic polymorphisms in drug-metabolizing enzyme genes and irinotecan-induced neutropenia during the first and second cycles of irinotecan-based treatment in 41 patients with mCRC. Analyses were conducted using the dominant genetic model, comparing individuals carrying at least one variant allele with those homozygous for the wild-type allele. (DOCX) [file pone.0338442.s001.docx]

**Supporting information**

**S1 Table. Impacts of polymorphisms in drug-metabolizing enzyme genes on irinotecan-induced neutropenia in the first and second cycle (Dominant Model) (n=41).**

| Gene | Genotype | n | Neutropenia | | | | | | | | | | | |
| --- | --- | --- | --- | --- | --- | --- | --- | --- | --- | --- | --- | --- | --- | --- |
|  |  |  | First Cycle | | | | | | Second Cycle | | | | | |
|  |  |  | Grade 0 | Grade 1-4 | *p* | Grade 0-2 | Grade 3-4 | *p* | Grade 0 | Grade 1-4 | *p* | Grade 0-2 | Grade 3-4 | *p* |
|  |  |  | n (%) | n (%) |  | n (%) | n (%) |  | n (%) | n (%) |  | n (%) | n (%) |  |
| *UGT1A1* | | | | | | | | | | | | | | |
| *28 ((TA)7TAA) | TA6/TA6 | 31 | 20(64.50) | 11 (35.50) | 0.075 | 27(87.10) | 4 (12.90) | 0.807 | 18 (56.70) | 13 (41.90) | 0.319 | 25(80.60) | 6 (19.40) | 0.964 |
|  | TA6/TA7+TA7/TA7 | 10 | 3(30.00) | 7 (70.00) |  | 9(90.00) | 1 (10.00) |  | 4(40.00) | 6 (60.00) |  | 8(80.00) | 2 (20.00) |  |
| *6 (211G>A) | G/G | 34 | 23(67.60) | 11 (32.40) | 0.00091* | 33(97.10) | 1 (2.90) | 0.0018* | 21(61.80) | 13 (38.20) | 0.022* | 30(88.20) | 4 (11.80) | 0.006* |
|  | G/A+A/A | 7 | 0(0.00) | 7 (100.00) |  | 3(42.90) | 4 (57.10) |  | 1(14.30) | 6 (85.70) |  | 3(42.90) | 4 (57.10) |  |
| *CYP3A4* | | | | | | | | | | | | | | |
| *1B (c.-392A>G) | A/A | 41 | 23 (56.10) | 18 (43.90) | N/A | 36(87.80) | 5 (12.20) | N/A | 22(53.70) | 19 (46.30) | N/A | 33(80.50) | 8 (19.50) | N/A |
| *18 (c.878T>C) | T/T | 40 | 12(55.00) | 18 (45.00) | 0.37 | 35(87.50) | 5 (12.50) | 0.706 | 21(52.50) | 19 (47.50) | 0.347 | 32(80.00) | 8 (20.00) | 0.618 |
|  | T/C+C/C | 1 | 1(100.00) | 0 (0.00) |  | 1(100.00) | 0 (0.00) |  | 1(100.00) | 0 (0.00) |  | 1(100.00) | 0 (0.00) |  |
| *CYP3A5* | | | | | | | | | | | | | | |
| *3 (c.6986A>G) | A/A | 6 | 4(66.70) | 2 (33.30) | 0.572 | 6(100.00) | 0 (0.00) | 0.323 | 3(50.00) | 3 (50.00) | 0.846 | 5(83.30) | 1 (16.70) | 0.849 |
|  | A/G+G/G | 35 | 19 (54.30) | 16 (45.70) |  | 30(85.70) | 5 (14.30) |  | 19 (54.30) | 16 (45.70) |  | 28(80.00) | 7 (20.00) |  |
| *CES1* | | | | | | | | | | | | | | |
| rs2244613 (c.1165-33C>A) | C/C | 17 | 10(58.80) | 7 (41.20) | 0.767 | 15(88.20) | 2 (11.80) | 0.943 | 9(52.90) | 8 (47.10) | 0.938 | 14(82.40) | 3 (17.60) | 0.823 |
|  | C/A+A/A | 24 | 13(54.20) | 11 (45.80) |  | 21(87.50) | 3 (12.50) |  | 13(54.20) | 11 (45.80) |  | 19(79.20) | 5 (20.80) |  |
| rs2244614 (c.1165-41G>A) | G/G | 24 | 13(54.20) | 11 (45.80) | 0.767 | 21(87.50) | 3 (12.50) | 0.943 | 14(58.30) | 10 (41.70) | 0.476 | 20(83.30 | 4 (16.70) | 0.585 |
|  | G/A+A/A | 17 | 10(58.80) | 7 (41.20) |  | 15(88.20) | 2 (11.80) |  | 8(47.10) | 9 (52.90) |  | 13(76.50) | 4 (23.50) |  |
| rs8192935 (c.257+885A>G) | A/A | 22 | 12(54.50) | 10 (45.50) | 0.829 | 19(86.40) | 3 (13.60) | 0.762 | 13 (59.10) | 9 (40.90) | 0.453 | 18(81.80) | 4 (18.20) | 0.817 |
|  | A/G+G/G | 19 | 11(57.90) | 8 (42.10) |  | 17(89.50) | 2 (10.50) |  | 9(47.40) | 10 (52.60) |  | 15(78.90) | 4 (21.10) |  |

Note. Genetic polymorphism associated with neutropenia in first and second cycle of irinotecan-base regimen treatment in 41 mCRC patients. N/A does not analyze, value with * indicate the statistically significant with Bonferroni-corrected (*p* value < 0.002), grades 1-4 was considered as toxicity and grades 3-4 was considered as severe toxicity.
