# Supplementary material for: Impacts of polymorphisms in drug-metabolizing enzyme and transporter genes on irinotecan toxicity and efficacy in Thai colorectal cancer patients
Source: PLoS One. 2025 Dec 12;20(12):e0338442. doi: 10.1371/journal.pone.0338442 (PMC12700395; doi:10.1371/journal.pone.0338442)
Supplement: S2 Table — This table summarizes the associations between genetic polymorphisms in drug-transporter genes and irinotecan-induced neutropenia during the first and second cycles of irinotecan-based treatment in 41 patients with mCRC. Analyses were conducted using the dominant genetic model, comparing individuals carrying at least one variant allele with those homozygous for the wild-type allele. (DOCX) [file pone.0338442.s002.docx]

**Supporting information**

**S2 Table. Impacts of polymorphisms in drug-transporter genes on irinotecan-induced neutropenia in the first and second cycle (Dominant Model) (n=41).**

| Gene | Genotype | n | Neutropenia | | | | | | | | | | | |
| --- | --- | --- | --- | --- | --- | --- | --- | --- | --- | --- | --- | --- | --- | --- |
|  |  |  | First Cycle | | | | | | Second Cycle | | | | | |
|  |  |  | Grade 0 | Grade 1-4 | *p* | Grade 0-2 | Grade 3-4 | *p* | Grade 0 | Grade 1-4 | *p* | Grade 0-2 | Grade 3-4 | *p* |
|  |  |  | n (%) | n (%) |  | N (%) | n (%) |  | n (%) | n (%) |  | n (%) | n (%) |  |
| *ABCB1* | | | | | | | | | | | | | | |
| rs1045642 (c.3435T>C) | C/C | 15 | 11(73.30) | 4 (26.70) | 0.114 | 14(93.30) | 1 (6.70) | 0.636 | 11(73.30) | 4 (26.70) | 0.103 | 15(100.00) | 0 (0.00) | 0.058 |
|  | C/T+T/T | 26 | 12(46.20) | 14 (53.80) |  | 22(84.60) | 4 (15.40) |  | 11(42.30) | 15 (57.70) |  | 18(69.20) | 8 (30.80) |  |
| rs1128503 (c.1236C>T) | C/C | 5 | 4(80.00) | 1 (20.00) | 0.363 | 5(100.00) | 0 (0.00) | 0.972 | 4(80.00) | 1 (20.00) | 0.350 | 5(100.00) | 0 (0.00) | 0.563 |
|  | C/T+T/T | 36 | 19(52.80) | 17 (47.20) |  | 31(86.10) | 5 (13.90) |  | 18(50.00) | 18 (50.00) |  | 28(77.80) | 8 (22.20) |  |
| rs2032582 (c.2677C>A) | C/C | 10 | 6(60.00) | 4 (40.00) | 0.775 | 9(90.00; | 1 (10.00) | 0.807 | 7(70.00) | 3 (30.00) | 0.233 | 9(90.00) | 1 (10.00) | 0.653 |
|  | C/A+A/A | 31 | 17(54.80) | 14 (45.20) |  | 27(87.10) | 4 (12.90) |  | 15(48.40) | 16 (51.60) |  | 24(77.40) | 7 (22.60) |  |
| rs2032582 (c.2677C>T) | C/C | 10 | 6(60.00) | 4 (40.00) | 0.775 | 9(90.00) | 1 (10.00) | 0.807 | 7(70.00) | 3 (30.00) | 0.233 | 9(90.00) | 1 (10.00) | 0.653 |
|  | C/T+T/T | 31 | 17(54.80) | 14 (45.20) |  | 27(87.10) | 4 (12.90) |  | 15(48.40) | 16 (51.60) |  | 24(77.40) | 7 (22.60) |  |
| *ABCG1* | | | | | | | | | | | | | | |
| rs225440 (c.286+7029C>T) | C/C | 20 | 10(50.00) | 10 (50.00) | 0.443 | 17(85.00) | 3 (15.00) | 0.592 | 11(55.00) | 9 (45.00) | 0.867 | 14(70.00) | 6 (30.00) | 0.131 |
|  | C/T+T/T | 21 | 13(61.90) | 8 (38.10) |  | 19(90.50) | 2 (9.50) |  | 11(52.40) | 10 (47.60) |  | 19(90.50) | 2 (9.50) |  |
| *ABCG2* | | | | | | | | | | | | | | |
| rs2231142 (c.421C>A) | C/C | 25 | 13(52.00) | 12 (48.00) | 0.509 | 22(88.00) | 3 (12.00) | 0.962 | 13(52.00) | 12 (48.00) | 0.791 | 19(76.00) | 6 (24.00) | 0.448 |
|  | C/A+A/A | 16 | 10(62.50) | 6 (37.50) |  | 14(87.50) | 2 (12.50) |  | 9(56.20) | 7 (43.80) |  | 14(87.50) | 2 (12.50) |  |
| rs2231137 (c.34G>A) | G/G | 13 | 8(61.50) | 5 (38.50) | 0.632 | 10(76.90) | 3 (23.10) | 0.147 | 7(53.80) | 6 (46.20) | 0.987 | 10(76.90) | 3 (23.10) | 0.692 |
|  | G/A+A/A | 28 | 15(53.60) | 13 (46.40) |  | 26(92.90) | 2 (7.10) |  | 15(53.60) | 13 (46.40) |  | 23(82.10) | 5 (17.90) |  |
| rs2622604 (c.1143C>T) | G/G | 25 | 13(52.00) | 12 (48.00) | 0.509 | 20(80.00) | 5 (20.00) | 0.056 | 12(48.00) | 13 (52.00) | 0.364 | 18(72.00) | 7 (28.00) | 0.086 |
|  | G/A+A/A | 16 | 10(62.50) | 6 (37.50) |  | 16(100.00) | 0 (0.00) |  | 10(62.50) | 6 (37.50) |  | 15(93.70) | 1 (6.30) |  |
| rs2231164 (c.1738-46A>G) | T/T | 11 | 6(54.50) | 5 (45.50) | 0.903 | 11(100.00) | 0 (0.00) | 0.148 | 7(63.60) | 4 (36.40) | 0.499 | 10(90.90) | 1 (9.10) | 0.412 |
|  | T/C+C/C | 30 | 17(56.70) | 13 (43.30) |  | 25(83.30) | 5 (16.70) |  | 15(50.00) | 15 (50.00) |  | 23(76.70) | 7 (23.30) |  |
| rs4148157 (c.1368-334C>T) | G/G | 22 | 10(45.50) | 12 (54.50) | 0.139 | 19(86.40) | 3 (13.60) | 0.762 | 9(40.90) | 13 (59.10) | 0.078 | 16(72.70) | 6 (27.30) | 0.177 |
|  | G/A+A/A | 19 | 13(68.40) | 6 (31.60) |  | 17(89.50) | 2 (10.50) |  | 13(68.40) | 6 (31.60) |  | 17(89.50) | 2 (10.50) |  |
| rs1871744 (c.690-217A>G) | A/A | 18 | 13(72.20) | 5 (27.80) | 0.066 | 16(88.90) | 2 (11.10) | 0.851 | 12(66.70) | 6 (33.30) | 0.139 | 16(88.90) | 2 (11.10) | 0.429 |
|  | A/G+G/G | 23 | 10(43.50) | 13 (56.50) |  | 20(87.00) | 3 (13.00) |  | 10(43.50) | 13 (56.50) |  | 17(73.90) | 6 (26.10) |  |
| *ABCC2* | | | | | | | | | | | | | | |
| rs3740066 (c.3927C>T) | C/C | 24 | 17(70.80) | 7 (29.20) | 0.024* | 20(83.30) | 4 (16.70) | 0.299 | 17(70.80) | 7 (29.20) | 0.009* | 21(87.50) | 3 (12.50) | 0.178 |
|  | C/T+T/T | 17 | 6(35.30) | 11 (64.70) |  | 16(94.10) | 1 (5.90) |  | 5(29.40) | 12 (70.60) |  | 12(70.60) | 5 (29.40) |  |
| rs717620 (c.-24C>T) | C/C | 27 | 18(66.70) | 9 (33.30) | 0.097 | 23(85.20) | 4 (14.80) | 0.645 | 18(66.70) | 9 (33.30) | 0.026* | 22(81.50) | 5 (18.50) | 0.824 |
|  | C/T+T/T | 14 | 5(35.70) | 9 (64.30) |  | 13(92.90) | 1 (7.10) |  | 4(28.60) | 10 (71.40) |  | 11(78.60; | 3 (21.40) |  |
| *ABCC5* | | | | | | | | | | | | | | |
| rs2292997 (c.129+7980C>T) | C/C | 20 | 9(45.00( | 11 (55.00) | 0.162 | 18(90.00) | 2 (10.00) | 0.675 | 9(45.00) | 11 (55.00) | 0.278 | 17(85.00; | 3 (15.00) | 0.477 |
|  | C/T+T/T | 21 | 14(66.70) | 7 (33.30) |  | 18(85.70) | 3 (14.30) |  | 13 (61.90) | 8 (38.10) |  | 16(76.20) | 5 (23.80) |  |
| *SLCO1B1* | | | | | | | | | | | | | | |
| rs4149056 (c.521T>C) | T/T | 33 | 18(54.50) | 15 (45.50) | 0.684 | 30(90.90) | 3 (9.10) | 0.217 | 18(54.50) | 15 (45.50) | 0.817 | 17(81.80) | 6 (18.20) | 0.662 |
|  | T/C+C/C | 8 | 5(62.50) | 3 (37.50) |  | 6(75.00) | 2 (25.00) |  | 4(50.00) | 4 (50.00) |  | 6(75.00) | 2 (25.00) |  |
| rs2306283 (c.388A>G) | A/A | 7 | 3(42.90) | 4 (57.10) | 0.679 | 7(100.00) | 0 (0.00) | 0.567 | 4(57.10) | 3 (42.90) | 0.839 | 5(71.40) | 2 (28.60) | 0.507 |
|  | A/G+G/G | 34 | 20(58.80) | 14 (41.20) |  | 29(85.30) | 5 (14.70) |  | 18(52.90) | 16 (47.10) |  | 28(82.40) | 6 (17.60) |  |

Note. Genetic polymorphism associated with neutropenia in first and second cycle of irinotecan-base regimen treatment in 41 mCRC patients. N/A does not analyze, value with * indicate the statistically significant with Bonferroni-corrected (*p* value < 0.002), grades 1-4 was considered as toxicity and grades 3-4 was considered as severe toxicity.
