# Supplementary material for: Impacts of polymorphisms in drug-metabolizing enzyme and transporter genes on irinotecan toxicity and efficacy in Thai colorectal cancer patients
Source: PLoS One. 2025 Dec 12;20(12):e0338442. doi: 10.1371/journal.pone.0338442 (PMC12700395; doi:10.1371/journal.pone.0338442)
Supplement: S3 Table — This table summarizes the associations between genetic polymorphisms in drug-metabolizing enzyme genes and treatment response rates in 41 patients receiving irinotecan-based therapy for mCRC. Analyses were conducted using the dominant genetic model, comparing individuals carrying at least one variant allele with those homozygous for the wild-type allele. (DOCX) [file pone.0338442.s003.docx]

**Supporting information**

**S3 Table. Impacts of polymorphisms in drug-metabolizing enzyme genes on response rates (Dominant Model) (n=41).**

| Gene | Genotype | n | Treatment Response | | |
| --- | --- | --- | --- | --- | --- |
|  |  |  | Non-responders (SD+PD) | Responders (CR+PR) | *p* |
|  |  |  | n (%) | n (%) |  |
| *UGT1A1* | | | | | |
| *28 ((TA)7TAA) | TA6/TA6 | 31 | 29 (93.50) | 2 (6.50) | 0.410 |
|  | TA6/TA7+TA7/TA7 | 10 | 10 (100.00) | 0 (0.00) |  |
| *6 (211G>A) | G/G | 34 | 32 (94.10) | 2 (5.90) | 0.511 |
|  | G/A+A/A | 7 | 7 (100.00) | 0 (0.00) |  |
| *CYP3A4* | | | | | |
| *1B (c.-392A>G) | A/A | 41 | 39 (95.10) | 2 (4.90) | N/A |
| *18 (c.878T>C) | T/T | 40 | 38 (95.00) | 2 (5.00) | 0.819 |
|  | T/C+C/C | 1 | 1 (100.00) | 0 (0.00) |  |
| *CYP3A5* | | | | | |
| *3 (c.6986A>G) | A/A | 6 | 6 (100.00) | 0 (0.00) | 0.548 |
|  | A/G+G/G | 35 | 33 (95.10) | 2 (5.70) |  |
| *CES1* | | | | | |
| rs2244613 (c.1165-33C>A) | C/C | 17 | 16 (94.10) | 1 (5.90) | 0.802 |
|  | C/A+A/A | 24 | 23 (95.80) | 1 (4.20) |  |
| rs2244614 (c.1165-41G>A) | G/G | 24 | 23 (95.80) | 1 (4.20) | 0.802 |
|  | G/A+A/A | 17 | 16 (94.10) | 1 (5.90) |  |
| rs8192935 (c.257+885A>G) | A/A | 22 | 21 (95.50) | 1 (4.50) | 0.915 |
|  | A/G+G/G | 19 | 18 (94.10) | 1 (5.30) |  |

Note. Genetic polymorphism is associated with the efficacy of irinotecan-based regimen treatment in 41 mCRC patients. N/A does not analyze, value with * indicate the statistically significant with Bonferroni-corrected (*p* < 0.002), non-responder was considered for stable disease (SD) and progressive (PD) disease and responder was considered for complete response (CR) and partial response (PR).
