# Supplementary material for: Impacts of polymorphisms in drug-metabolizing enzyme and transporter genes on irinotecan toxicity and efficacy in Thai colorectal cancer patients
Source: PLoS One. 2025 Dec 12;20(12):e0338442. doi: 10.1371/journal.pone.0338442 (PMC12700395; doi:10.1371/journal.pone.0338442)
Supplement: S4 Table — (Dominant Model). This table summarizes the associations between genetic polymorphisms in drug-transporter genes and treatment response rates in 41 patients receiving irinotecan-based therapy for mCRC. Analyses were conducted using the dominant genetic model, comparing individuals carrying at least one variant allele with those homozygous for the wild-type allele. (DOCX) [file pone.0338442.s004.docx]

**Supporting information**

**S4 Table. Impacts of polymorphisms in drug transporter genes on response rates
(Dominant Model) (n=41).**

| Gene | Genotype | n | Treatment Response | | |
| --- | --- | --- | --- | --- | --- |
|  |  |  | Non-responders (SD+PD) | Responders (CR+PR) | *p* |
|  |  |  | n (%) | n (%) |  |
| *ABCB1* | | | | | |
| rs1045642 (c.3435T>C) | C/C | 15 | 15 (100.00) | 0 (0.00) | 0.271 |
|  | C/T+T/T | 26 | 24 (92.30) | 2 (7.70) |  |
| rs1128503 (c.1236C>T) | C/C | 5 | 5 (100.00) | 0 (0.00) | 0.589 |
|  | C/T+T/T | 36 | 34 (94.40) | 2 (5.60) |  |
| rs2032582 (c.2677C>A) | C/C | 10 | 9 (90.00) | 1 (10.00) | 0.387 |
|  | C/A+A/A | 31 | 30 (96.80) | 1 (3.20) |  |
| rs2032582 (c.2677C>T) | C/C | 10 | 9 (90.00) | 1 (10.00) | 0.387 |
|  | C/T+T/T | 31 | 30 (96.80) | 1 (3.20) |  |
| *ABCG1* | | | | | |
| rs225440 (c.286+7029C>T) | C/C | 20 | 19 (95.00) | 1 (5.00) | 0.972 |
|  | C/T+T/T | 21 | 20 (95.20) | 1 (4.80) |  |
| *ABCG2* | | | | | |
| rs2231142 (c.421C>A) | C/C | 25 | 23 (92.00) | 2 (8.00) | 0.246 |
|  | C/A+A/A | 16 | 16 (100.00) | 0 (0.00) |  |
| rs2231137 (c.34G>A) | G/G | 13 | 12 (92.30) | 1 (7.70) | 0.569 |
|  | G/A+A/A | 28 | 27 (96.40) | 1 (3.60) |  |
| rs2622604 (c.1143C>T) | G/G | 25 | 23 (92.00) | 2 (8.00) | 0.246 |
|  | G/A+A/A | 16 | 16 (100.00) | 0 (0.00) |  |
| rs2231164 (c.1738-46A>G) | T/T | 11 | 11 (100.00) | 0 (0.00) | 0.380 |
|  | T/C+C/C | 30 | 28 (93.30) | 2 (6.70) |  |
| rs4148157 (c.1368-334C>T) | G/G | 22 | 20 (90.90) | 2 (9.10) | 0.178 |
|  | G/A+A/A | 19 | 19 (100.00) | 0 (0.00) |  |
| rs1871744 (c.690-217A>G) | A/A | 18 | 18 (100.00) | 0 (0.00) | 0.201 |
|  | A/G+G/G | 23 | 21 (91.30) | 2 (8.70) |  |
| *ABCC2* | | | | | |
| rs3740066 (c.3927C>T) | C/C | 24 | 23 (95.80) | 1 (4.20) | 0.802 |
|  | C/T+T/T | 17 | 16 (94.10) | 1 (5.90) |  |
| rs717620 (c.-24C>T) | C/C | 27 | 24 (92.60) | 2 (7.40) | 0.296 |
|  | C/T+T/T | 14 | 14 (100.00) | 0 (0.00) |  |
| *ABCC5* | | | | | |
| rs2292997 (c.129+7980C>T) | C/C | 20 | 19 (95.00) | 1 (5.00) | 0.972 |
|  | C/T+T/T | 21 | 20 (95.20) | 1 (4.80) |  |
| *SLCO1B1* | | | | | |
| rs4149056 (c.521T>C) | T/T | 33 | 31 (93.90) | 2 (6.10) | 0.475 |
|  | T/C+C/C | 8 | 8 (100.00) | 0 (0.00) |  |
| rs2306283 (c.388A>G) | A/A | 7 | 6 (85.70) | 1 (14.30) | 0.204 |
|  | A/G+G/G | 34 | 33 (97.10) | 1 (2.90) |  |

Note. Genetic polymorphism is associated with the efficacy of irinotecan-based regimen treatment in 41 mCRC patients. N/A does not analyze, value with * indicate the statistically significant with Bonferroni-corrected (*p* < 0.002), non-responder was considered for stable disease (SD) and progressive (PD) disease and responder was considered for complete response (CR) and partial response (PR).
